# Fatigue, depression, and product tolerability during long-term treatment with intravenous immunoglobulin (Gamunex® 10%) in patients with chronic inflammatory demyelinating polyneuropathy

**DOI:** 10.1186/s12883-023-03223-5

**Published:** 2023-05-26

**Authors:** Juliane Klehmet, Björn Tackenberg, Judith Haas, Bernd C. Kieseier

**Affiliations:** 1 Charité - Universitätsmedizin Berlin, Neurocure Clinical Research Center Berlin, Charitéplatz 1, 10117 Berlin, Germany; 2 Jüdisches Krankenhaus Berlin, Heinz-Galinski-Straße 1, 13347 Berlin-Mitte, Germany; 3 Klinik Und Poliklinik Für Neurologie, Baldingerstrasse 1, 35043 Marburg, Germany; 4 Klinik Fur Neurologie, Heinrich-Heine Universität, Moorenstrasse 5, 40225 Düsseldorf, Germany

**Keywords:** CIDP, IVIG, Real-world evidence, Patient-reported outcome measures, Quality of life

## Abstract

**Abstract:**

**Introduction/Aims:**

Chronic inflammatory demyelinating polyradiculoneuropathy (CIDP) is characterized by progressive weakness and sensory loss, often affecting patient’s ability to walk and perform activities of daily living independently. Furthermore, patients often report fatigue and depression which can affect their quality of life. These symptoms were assessed in CIDP patients receiving long-term intravenous immunoglobulin (IVIG) treatment.

**Methods:**

GAMEDIS was a multi-center, prospective, non-interventional study in adult CIDP patients treated with IVIG (10%) and followed for two years. Inflammatory Neuropathy Cause and Treatment (INCAT) disability score, Hughes Disability Scale (HDS), Fatigue Severity Scale (FSS), Beck Depression Inventory II (BDI), Short Form-36 health survey (SF-36) and Work Productivity and Activity Impairment Score Attributable to General Health (WPAI-GH) were assessed at baseline and quarterly. Dosing and treatment intervals, changes in outcome parameters, and adverse events (AEs) were analyzed.

**Results:**

148 evaluable patients were followed for a mean of 83.3 weeks. The mean maintenance IVIG dose was 0.9 g/kg/cycle (mean cycle interval 38 days). Disability and fatigue remained stable throughout the study. Mean INCAT score: 2.4 ± 1.8 at baseline and 2.5 ± 1.9 at study end. HDS: 74.3% healthy/minor symptoms at baseline and 71.6% at study end. Mean FSS: 4.2 ± 1.6 at baseline and 4.1 ± 1.7 at study end. All patients reported minimal/no depression at baseline and throughout. SF-36 and WPAI-GH scores remained stable. Fifteen patients (9.5%) experienced potentially treatment-related AEs. There were no AEs in 99.3% of infusions.

**Discussion:**

Long-term treatment of CIDP patients with IVIG 10% in real-world conditions maintained clinical stability on fatigue and depression over 96 weeks. This treatment was well-tolerated and safe.

## Introduction

Chronic inflammatory demyelinating polyradiculoneuropathy (CIDP) is a rare and often disabling chronic progressive or relapsing immune-mediated neuropathy. The prevalence reported in the literature varies between 0.67 and 7.7 cases per 100,000 persons [1]. Typical CIDP is clinically characterised by symmetric proximal and distal weakness and sensory dysfunction of all extremities, developing over at least two months with decreased tendon reflexes [2, 3]. Disease presentation can diverge, with some patients atypically presenting with subacute to chronically progressive symptoms which may be asymmetric, focal, pure motor or pure sensory [2–6]. Weakness and accompanying fatigue greatly interfere with daily occupational and social activities and may have a profound impact on patients’ quality of life, which may result in depression [7, 8].

Corticosteroids, intravenous immunoglobulin (IVIG), and plasma exchange are recommended first-line treatments for CIDP [9]. While all treatment options are generally considered equally effective [10–12], current clinical evidence suggests that IVIG is better tolerated than other therapies, especially during long-term treatment [13, 14]. Moreover, IVIG treatment provides a faster onset of efficacy than corticosteroids and is less invasive than plasma exchange [15].

The *Immune Globulin Intravenous CIDP Efficacy* (ICE) trial, a randomized, double-blind, placebo-controlled, response-conditional crossover trial in 117 CIDP patients showed that IVIG (Gamunex® 10%) was superior to placebo based on results from the adjusted Inflammatory Neuropathy Cause And Treatment (INCAT) disability score, and grip strength measures [16]. IVIG treatment was also associated with an improvement in patients’ quality of life (QoL), based on 36-Item Short Form Survey (SF-36) domains during the first 24 weeks of treatment. QoL was generally increased or maintained in patients re-assigned to IVIG during the extension phase of the trial (up to 48 weeks) [6].

Chronic fatigue is a common symptom in immune-mediated polyneuropathies and is known to negatively impact patients’ QoL. Chronic fatigue can persist for years in CIDP patients [17]. Likewise, a chronic condition such as CIDP can adversely influence patient’s mental health and patients may develop depression [4].

While it has been shown that IVIG improves disability in the clinical trial setting, few studies have systematically documented the long-term effects of IVIG therapy on fatigue and depression in CIDP patients in a real-world setting [12, 18].

Hence, the objective of this observational, multi-centre study was to describe the influence of Gamunex® 10% IVIG on fatigue and depression dynamics over time and to confirm tolerability and safety in real-world conditions.

## Materials and methods

### Design

The observational GAMEDIS-2 study was a prospective, open, non-interventional, multi-center study conducted in Germany between November 2012 and February 2016. The participating physicians were neurologists working in hospitals or office-based. The observational period lasted up to 96 weeks, including the baseline visit and quarterly follow-up observations. Due to the non‐interventional nature of the study, the treatment of patients did not follow a strictly predetermined protocol, and only procedures and results arising from routine care were documented. Patients were required to complete patient-reported outcomes questionnaires (PROs) addressing fatigue, depression, quality of life, work productivity, and activity impairment during routine on-site clinic visits.

The study protocol was approved by the ethics committee of the Charité University Hospital (Berlin, Germany). This study was carried out in compliance with an observational plan, regulatory requirements, and the ethical principles of the latest revision of the Declaration of Helsinki as adopted by the World Medical Association [19]. The study was conducted in accordance with the German Medicinal Products Act and followed the recommendations of the Paul-Ehrlich-Institute and the German Federal Institute for Drugs and Medical Devices.

### Study population

Patients aged 18 years or over who were diagnosed with CIDP according to the European Federation of Neurological Societies (EFNS)/Peripheral Nerve Society (PNS) consensus guidelines [20]. These guidelines require a combination of clinical and electrodiagnostic tests showing peripheral nerve demyelination with exclusions to eliminate other disorders that may present similarly to CIDP. Objective assessment of other endpoints including laboratory abnormalities (elevated cerebrospinal fluid protein with leukocyte count < 10/mm^3^) magnetic resonance imaging, nerve biopsy, and an immunotherapy trial could assist the diagnosis as supportive criteria. Patients who provided written, informed consent to participate were eligible for the study.

Patients could be IVIG-naïve, treated with IVIG in the past, or currently on IVIG (Gamunex® 10%. Grifols, Barcelona, Spain). Gamunex® 10% was prescribed and administered in accordance with the approved labelling.

Baseline measures included patient demographics (e.g., age, sex, body mass index [BMI]), presence of co-morbidities, concomitant medications, date of CIDP diagnosis and CIDP treatment history.

### Outcome measures

At baseline and each quarterly visit, patient’s disability status was measured by physicians using INCAT score [10] and the Hughes Disability Scale (HDS) [21]. Additionally, patients filled in PROs to assess fatigue (Fatigue Severity Scale-FSS) [22], depression (Beck Depression Inventory II- BDI-II) [23], quality of life (SF-36 questionnaire) and Work Productivity and Activity Impairment Attributable to General Health (WPAI-GH) [24] at the same time points. The mean scores the SF-36 questionnaire were compared to a healthy reference population [25].

The INCAT disability score was used to measure activity limitations. It is frequently used as a primary endpoint in inflammatory polyneuropathy clinical trials [26]. The INCAT is comprised of two parts: the arm score and the leg score. Each part is scored between 0 and 5 points, resulting in an INCAT total score between 0 and 10 with higher scores meaning greater disability. The HDS is a functional rating scale for autoimmune neuropathy with scores ranging from 0 (“healthy”) to 6 (“death”).

The FSS, a 9-item self-reporting questionnaire, measures fatigue severity and the impact of fatigue on a person's activities and lifestyle. Responses are scored on a 7-point scale (1 = strongly disagree to 7 = strongly agree). FSS score < 3 indicates no or very low fatigue, between 3 and 5 moderate fatigue, and > 5 severe fatigue.

The BDI questionnaire provides a quantitative assessment of the intensity of depression [23]. Because it is designed to reflect the depth of depression, it can monitor changes over time and provide an objective measure for judging improvement. It consists of 21 questions; each answer being scored on a scale of 0 to 3. Higher total scores indicate more severe depressive symptoms. Patients with a score below 14 are classified as “minimally depressed.” Scores between 14 and 19 indicate “mild depression”, 20 to 28 indicates “moderate depression”, and scores equal or greater than 29 indicate “severe depression.” If more than two questions were not answered, the BDI was not calculated.

The WPAI-GH questionnaire [24] quantifies the number of hours that the respondent is unable to work and evaluates the extent to which their general health state affects their productivity while working. In detail, absenteeism (percentage of work time missed due to general health), presenteeism (percentage of impairment while working due to general health), and overall work impairment (percentage of overall work impairment due to general health) were calculated in employed patients. Additionally, activity impairment (percentage of general activity impairment due to general health) was calculated for all patients. All scores range between 0 and 100%, with higher scores indicating greater impairment and reduced productivity. In case of missing items, the respective scores were not evaluable.

The SF-36 health survey measures an individual’s overall subjective health status [27]. It consists of 36 items assessing eight dimensions of the health status (physical functioning, physical role functioning, bodily pain, general health perceptions, vitality, social role functioning, emotional role functioning, and mental health). Each score ranges between 0 and 100 with higher score values indicating a better health state. At least half of the items of one dimension had to be available to be considered evaluable.

### Treatment data

During the study, patients were treated with Gamunex® 10% according to the German Summary of Product Characteristics (SmPC) for CIDP [28] which recommends an induction dose of 2 g/kg administered over two to four consecutive days (i.e. several infusions within one treatment cycle) and a maintenance dose of 1 g/kg over one day or two consecutive days every three weeks. However, according to the non-interventional design of the study, physicians were free to choose alternate dosage regimens at their own discretion, which is also explicitly allowed by the SmPC [28]. Infusion data (dose administered per infusion) were collected throughout the study period.

### Safety and tolerability

The safety of Gamunex® 10% was assessed through spontaneously reported adverse events. It was the responsibility of the investigator to document all potentially treatment-related adverse events (PTRAEs) which occurred during the study.

PTRAEs attributed to Gamunex® 10% were queried on the case report form of each visit and recorded in a Safety Report Form according to the "Medical Dictionary for Regulatory Activities" (MedDRA®, current version) preferred terms. Analysis by MedDRA® primary system organ class, absolute and relative frequencies of PTRAEs and the seriousness classification of the PTRAE ("serious" or "non-serious") were recorded.

### Data analysis and calculations

All enrolled patients who fulfilled the inclusion criteria and had available data for at least two visits were included in the analyses of baseline characteristics, outcome measures and treatment data. Safety and tolerability were assessed in all enrolled patients.

The total administered dose per treatment cycle was calculated as the sum of daily administered doses (g/kg) within one treatment cycle. Patient-specific averages were calculated for the total administered dose across all maintenance cycles (excluding the first cycle in patients without prior IVIG treatment), the dose per infusion across all maintenance cycles, and the number of infusions per maintenance cycle. Additionally, total dose, dose per infusion, and number of infusions for the first treatment cycle were described as the loading dose for IVIG-naïve patients.

In this non-interventional setting, the documentation interval of three months was only an estimate. Therefore, for the analysis, actual observations were assigned to quarterly time windows, and all observations taking place ± 41 days from the quarterly time point were grouped together. If two different observations took place within same time window, only the data of the observation closest to the quarterly time point was considered analysis.

Descriptive statistics were conducted for quantitative and qualitative variables for all patients and separately for patients with and without prior IVIG treatment. Additionally, the clinical outcomes were described for each quarterly visit.

## Results

### Demographic data and clinical characteristics

**Fig. 1:**
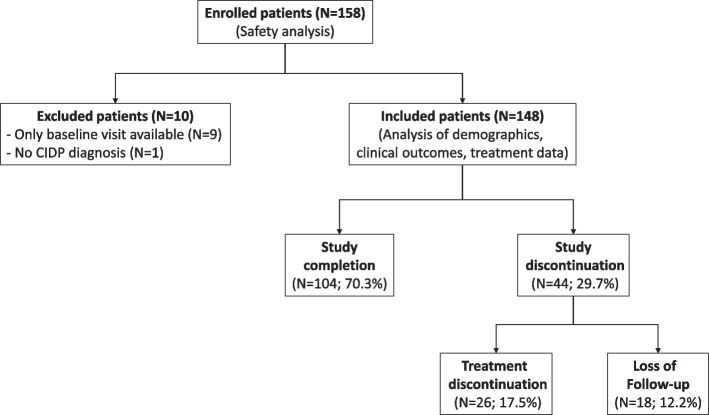
Flowchart of enrollment, treatment discontinuation, and study completion. CIDP = chronic inflammatory demyelinating polyneuropathy

In total, 158 patients were enrolled in the study by 46 neurologists (22 office-based and 24 hospital-based). For nine patients, only the baseline visit was available, and for one patient CIDP diagnosis was not confirmed. Although safety and tolerability data were reported for all 158, only 148 patients were included in the statistical analyses of demographic data, clinical characteristics, and treatment data. In total, 70% of the patients completed the study and less than 20% discontinued the treatment during the study period (Fig. 1).

Table 1 provides an overview of demographic and baseline characteristics. Out of 148 patients, 128 (86.5%) were already on IVIG therapy or had received IVIG in the past (IVIG pre-treated). About two thirds of the total patient population (66.9%) were male. The total study population had a median age of 65 years and a median BMI of 26.5 kg/m^2^. Median time since CIDP diagnosis was 43.6 months and was longer in patients with prior IVIG treatment compared to those receiving IVIG for the first time (46.2 vs 25.3 months). At baseline, 69 patients (46.6%) had already been treated with systemic steroids, and 31 (20.9%) with immunosuppressants. None of the patients in the study received concomitant immunosuppressive treatment.

**Table 1:** Demographic and baseline characteristics of patients

	**All patients**	**Previous IVIG treatment**	
		**Yes**	**No**
Patients, n (%)	148 (100)	128 (86.5)	20 (13.5)
Female/Male, n (%)	49/99 (33.1/66.9)	43/85 (33.6/66.4)	6/14 (30/70)
Age (years), median (min–max)	65 (24.0–89.0)	65 (24.0–89.0)	61 (41.0–80.0)
BMI (Kg/m^2^), median (min–max)	26.5 (17.9–43.1)	26.5 (17.9–43.1)	26.0 (18.6–36.0)
CIDP duration^a^ (months), median (min–max)	43.6 (0.1–327.1)	46.2 (1.7–327.1)	25.3 (0.1–181-7)
Baseline INCAT score, mean ± SD	2.4 ± 1.8	2.4 ± 1.8	2.6 ± 2.1
Baseline Hughes score, mean ± SD	1.3 ± 0.9	1.2 ± 0.9	1.6 ± 0.8
Baseline FSS score, mean ± SD	4.2 ± 1.6	4.2 ± 1.6	4.1 ± 1.6
Baseline BDI score, mean ± SD	10.9 ± 8.6	11.5 ± 8.8	6.5 ± 4.3

^a^time since CIDP diagnosis. *IVIG*  Intravenous immunoglobulin, *BMI* Body mass index, *CIDP* Chronic inflammatory demyelinating polyneuropathy, *INCAT*  Inflammatory Neuropathy Cause and Treatment disability score, *FSS* Fatigue Severity Scale, *BDI* Beck Depression Inventory II

The total patient population displayed a mean INCAT disability and a mean HDS functional score of 2.4 and 1.3 respectively. The mean FSS score was 4.2 and the mean BDI score was 9.0. These parameters were very similar in the IVIG-pretreated and IVIG-naïve patient populations, but for the mean BDI score in the IVIG-naïve population which was 6.5.

The percentage of the total population with a comorbidity was 53.4% (79/148), while 55.5% (71/128) of IVIG-pre-treated patients and 40.0% (8/20) of IVIG-naïve patients suffered from any comorbidity. Common comorbidities in the total population included arterial hypertension, type I or type II diabetes mellitus, adiposity or osteoporosis among others. Arterial hypertension being the most common (48.6%).

### Clinical outcomes

**Fig. 2:**
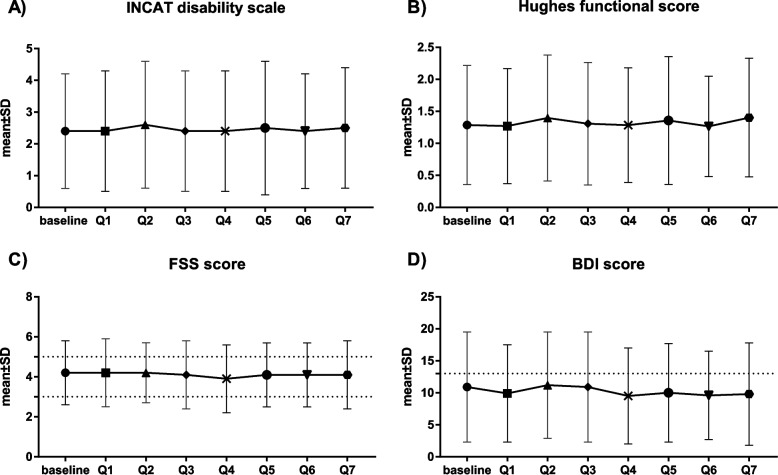
Mean scores of clinical outcome variables (± SD), INCAT (**A**), Hugues (**B**), FSS (**C**) and BDI (**D**) for each quarterly visit. INCAT = Inflammatory Neuropathy Cause and Treatment disability score; Hughes = Hughes Disability Score; FSS = Fatigue Severity Scale; BDI = BDI = Beck Depression Inventory II

Figure 2 shows the dynamics of total INCAT disability, HDS functional, FSS, and BDI scores (± standard deviation [SD]) over time. The mean INCAT disability score at baseline was 2.4 (± 1.8) and did not change at the quarterly observations (Fig. 2 A). Similarly, the mean HDS score remained stable. (Fig. 2 B).

### Patient-reported outcome measures

The mean FSS scores remained stable between 3 and 5 for all quarterly visits, reflecting moderate fatigue (Fig. 2 C). While the percentage of patients suffering from severe fatigue (FSS > 5) did not change from baseline to Q7 (34.6% to 34.3%), the percentage of patients with no or very low fatigue (FSS < 3) increased from 23.7% to 29.4% and the percentage of patients with moderate fatigue (FSS 3–5) diminished (41.7% to 36.3%).

The mean BDI scores were ≤ 14 (minimal depression) at all quarterly visits (Fig. 2 D). The percentage of patients reporting minimal depression increased from 68.8% at baseline to 75.5% at the end of the observational period.

**Fig. 3:**
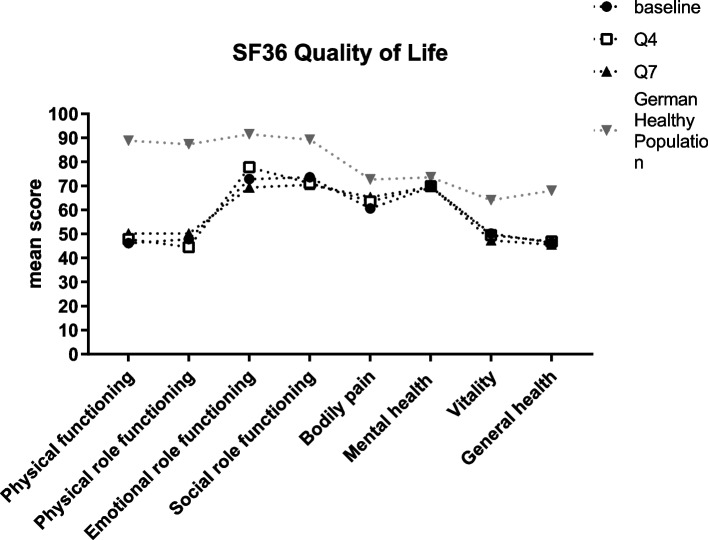
Mean scores of quality of life (SF-36 score): SF-36 = Short Form 36 health survey. Q4 = quarter 4 study visit; Q7 = quarter 7 study visit

As shown in Fig. 3, in general, all physical and mental component summary scores of the SF-36 remained stable across the 96-week follow-up. When compared with German general population SF-36 mean values, mean SF-36 physical and physical-role functioning scores in the CIDP study population were substantially lower compared with the normative data. There was however a slight shift towards SF-36 normal values for such physical domains. The rest of SF-36 domains of patients were closer to normative values and remained stable over time.

**Fig. 4:**
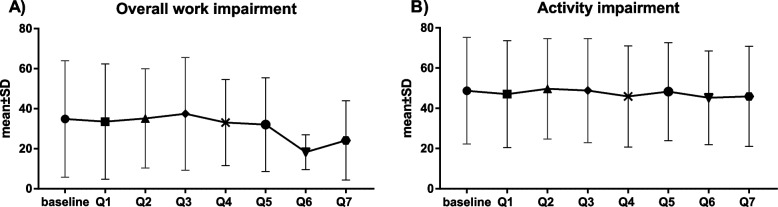
Mean scores (± SD), of overall work impairment (**A**) and activity impairment (**B**) at baseline and quarterly study visits (Q1-Q7).

The mean WPAI-GH values for overall work impairment in the employed population (27 patients [18.2%] employed patients at baseline) showed a minor decrease across the study (Fig. 4A). However, the activity impairment in the whole patient population (both unemployed and employed) remained stable over the course of the study (Fig. 4B).

### Treatment characteristics

Complete treatment records were available for 81 of 148 patients (10 of whom had no prior IVIG treatment). In total, 1554 infusions in 1148 treatment cycles were documented. Each patient was treated with a mean of 19.2 infusions.

The mean maintenance dose was 0.9 g/kg and was administered at a mean interval course of 38 days in a mean of 1.7 infusions per cycle (Table 2). The mean dose per infusion was 0.6 g/kg. The ten patients without prior IVIG treatment (IVIG-naïve) received a mean dose of 1.2 g/kg in the first documented cycle (loading dose).

**Table 2:** Treatment characteristics

Parameter	Value
Patients with complete records, n	81
Total number of infusions, n	1554
Number of infusions per patient, mean ± SD	19.2 ± 2.2
Maintenance dose (g/kg), mean ± SD	0.9 ± 0.5
Loading dose (IVIG-naive) (g/kg), mean ± SD	1.2 ± 0.5
Dose per infusion (g/kg), mean ± SD	0.6 ± 0.3
Number of infusion days per cycle. mean ± SD	1.7 ± 1.3
Treatment cycle interval (days), mean ± SD	38.1 ± 32.9

*IVIG* Intravenous immunoglobulin

### Safety and tolerability

In total, 34 PTRAEs were reported in 15 of the 158 enrolled patients (9.5%). The most common PTRAEs were headaches (*n* = 5). A very small number of infusions (6 of 1554: 0.4%) resulted in serious individual case safety reports (ICSRs). Four of these included well-known complications of IVIG treatment (e.g., hypersensitivity reactions, anaphylactic reactions, deep vein thrombosis, or central nervous system infarctions). The two-remaining serious ICSRs (atrial fibrillation, congestive cardiac failure, chest pain, increased blood pressure and creatine phosphokinase, and right bundle branch block) were considered unexpected, and possibly also related to the underlying coronary heart diseases of the respective patients.

## Discussion

The results of this study on long-term treatment of CIDP patients with Gamunex® 10% IVIG in real-world conditions showed clinical stability over time. The patient dropout observed in our study was 30%, which is the dropout average rate out of clinical trials [29]. Both disability measures and a broad range of PROs such as fatigue, depression and quality of life, remained stable over the 96-week follow up period.

Fatigue is a common symptom in CIDP and may persist in patients in disease remission [30]. A moderate level of fatigue was reported in 41.7% of patients in the GAMEDIS study at baseline, while 36.5% reported severe fatigue. The percentage of patients with severe fatigue in this study is in line with the frequency of patients that experienced severe fatigue in other studies: 38% to 74% [4, 31, 32]. The percentages of patients displaying low, moderate or severe fatigue did not change considerably from baseline to Q7 and mean FSS score remained stable during the 96 weeks of observation indicating that fatigue symptoms were well controlled under Gamunex® 10% treatment.

Fatigue is regarded by CIDP patients as one of their most disabling symptoms and it has a negative impact on their quality of life [17]. Recent studies have investigated the relationship between fatigue and different clinical parameters in CIDP patients and concluded that fatigue is associated with lower functional and quality of life outcomes [30, 33]. Although the GAMEDIS study design did not allow to analyse the influence of fatigue neither in disability nor in QoL, it is interesting to observe that the mean FSS scores, INCAT, HDS, QoL dimensions and WPAI-GH scores displayed similar dynamics and remained stable over time.

Depression is often reported by CIDP patients but in contrast to fatigue it is less common in patients in remission [30]. 68% of patients in the GAMEDIS study had minimal depression (BDI < 14) at baseline and the percentage increased to 75.5% at Q7. The mean BDI score remained below 14 and stable at all quarterly visits, which suggests that Gamunex® 10% treatment kept most patients with minimal depression and it even improved in some of them.

The mean maintenance dose used in this study was 0.9 g/kg at a mean cycle interval of 38 days. It is a slightly lower maintenance dose and a longer treatment interval than in the ICE trial [16]. Other reports have also shown that in clinical practice, CIDP patients are treated with different IVIG maintenance schedules [34]. In fact, the current European Academy of Neurology (EAN)/PNS guidelines, the European Medicines Agency SmpC for IVIGs, and the Gamunex® 10% German SmpC recommend that if IVIG treatment is effective, long term treatment should be subject to the physician’s discretion based upon the patient response and maintenance response, and that the dosing and intervals may have to be adapted according to the individual course of the disease [28, 35, 36]. Thus, the mean maintenance dosing and treatment intervals of Gamunex® 10% in this real-world study would be in line with the current maintenance treatment recommendations and suggest that physicians attempt to individualize IVIG treatment in CIDP patients whose disease is well controlled.

Improving IVIG dosing and treatment schedule are nowadays of great interest among the scientific community [37] and various randomized clinical trials are evaluating different IVIG dosages and schedules during CIDP maintenance treatment [38].

The ten IVIG naïve-patients in this study received a lower induction dose than the 2 g/kg used in the ICE trial and recommended in the Gamunex® 10% German SmPC and in the EAN/PNS guideline [35, 36]. Interestingly, a survey among CIDP-treating neurologists in the United States, highlighted that most physicians often administered IVIG lower doses and at longer intervals, not only as maintenance treatment but also as induction dose, again suggesting that they attempt to individualize IVIG treatment [39].

With respect to the safety profile, the PTRAEs rate we observed was only 9.5%, markedly lower than in ICE trial (34%) and the IMCT trial (46%) [13, 16]. This is not unexpected, since AEs recording in observational studies is less restrictive than in prospective clinical trials. Overall, this study confirms previous reports on the safety and good tolerability of long-term treatment with Gamunex® 10% and in line with safety profile described in the SmpC [28].

Limitations of this study include the non-interventional design that did not provide further information on the rationale of physicians’ treatment decisions. Because of the non-interventional nature of the study, the treatment of patients did not follow a strict predetermined protocol. Therefore, the time elapsed between patients’ diagnoses and their enrolment into the study varied widely (from 0.1 to 327.1 months). Moreover, despite the worldwide acceptance and use in research of the EFNS/PNS guideline with very good diagnostic accuracy [40, 41], misdiagnosis of CIDP may occur [42, 43]. In addition, descriptive statistics were used. However, this observational study was not intended to find out statistically significant differences but to describe the long-term evolution of fatigue and depression profiles in CIDP patients treated with a specific IVIG product, as well as its tolerability. Besides, the HDS, FSS and INCAT scales used in this study may not be perfect to assess CIDP patient’s fatigue and depression status. Future studies should explore how neurologists make treatment individualization decisions in real world practice.

In conclusion, long-term CIDP treatment with Gamunex® 10% IVIG in real-world conditions maintained clinical stability of patients as well as patient reported outcome measures over a 96-week follow up period and showed good tolerability and safety records.

## Data Availability

Data reported in this manuscript are available within the article. Additional data from the GAMEDIS-2 study are available from the corresponding author upon reasonable request.

## Abbreviations

AEs: Adverse events
BDI: Beck Depression Inventory II
BMI: Body mass index
CIDP: Chronic inflammatory demyelinating polyneuropathy
EAN: European Academy of Neurology
EFNS: European Federation of Neurological Societies
FSS: Fatigue Severity Scale
HDS: Hughes Disability Scale
INCAT: Inflammatory Neuropathy Cause and Treatment disability score
IVIG: Intravenous immunoglobulin
PNS: Peripheral Nerve Society
PROs: Patient-reported outcomes questionnaires
PTRAEs: Potentially treatment-related adverse events
QoL: Quality of life
SF-36: Short form-36 health survey
SmpC: Summary of Product Characteristics
WPAI-GH: Work Productivity and Activity Impairment Score Attributable to General Health
